# Phytoplankton and Its Role in Accumulation of Microelements in Bottom Deposits of Azov Sea

**DOI:** 10.1155/2019/8456371

**Published:** 2019-03-12

**Authors:** Irina V. Dotsenko, Anna V. Mikhailenko

**Affiliations:** Southern Federal University, Institute of Earth Science, Rostov-on-Don 344090, Russia

## Abstract

The importance of phytoplankton is high in transformation of substances in aquatic ecosystems and in formation of suspension's material structure. Its main functions are consumption of the dissolved biogenic components and chemical elements and their conversion to a firm phase. The article is devoted to the bioaccumulation of microelements by phytoplankton in the ecosystem of the Azov Sea. The fact that the algal biomass during the periods of blooming in the sea reaches 1,400 g/l makes this study especially urgent. The authors define the rates of biogeochemical cycle and the intensity of chemical elements' consumption and also investigate the role of phytoplankton in the formation chemical peculiarities of bottom deposits and its involvement in sedimentogenesis in the Azov Sea. The cause of the reduced trace element content in bottom deposits relatively to suspended material is established. It is noted that the amount of some elements annually consumed by algae of the Azov Sea is up to 75% from their maximum delivery by terrigenous material.

## 1. Introduction

The organic world plays an important role in the formation of chemical peculiarities of bottom deposits of epeiric seas of the arid belt. Such seas are characterized by high productivity of the entire ecosystem, and the Azov Sea is a typical example. Phytoplankton fulfilling a mobilizing function and enhancing geochemical mobility promotes separation of some chemical elements from others. Such a phytogenic selection determines their further differentiation at the stages of sedimentogenesis (synsedimentary processes) and diagenesis (postsedimentary processes). Different degrees of metal consumption can be an indirect evidence of the possibility of element extraction from suspended matter. Various degrees of microelement consumption can be an indirect evidence of extraction possibility from suspended matter [1–5]. Complex studies of various phases of marine sedimentogenesis imply the relevant processes in the water layer began rather recently [6, 7]. The studied chemical compounds have been found in marine and oceanic ecosystems for about forty years ago. Over the last years, a new phase of such studies has begun, that is due to the interest in studying bioavailability of microelements [8–11].

Strakhov [12] rightly called the processing of less labile compounds of elements that had come from the continent into more mobile organic compounds as the main geochemical function of biota. It is quite obvious that phytoplankton, which is the main producer of organic matter and the most powerful link of marine ecosystems, takes the greatest biogeochemical significance at the stage of marine sedimentogenesis [13]. Algae with their huge geochemical energy play leading role in the biogenic cycling of chemical elements being responsible also for various biogeochemical functions. The calculations carried out by Datsko [14] showed that the turnover ratio of nutrients (the ratio between the annual phytoplankton's consumption of one or another chemical element and its amount in dissolved form in the photosynthesis zone and in the main sources of nutrition) in the Azov Sea during the growing period reaches 8.

## 2. Materials and Methods

**Figure 1:**
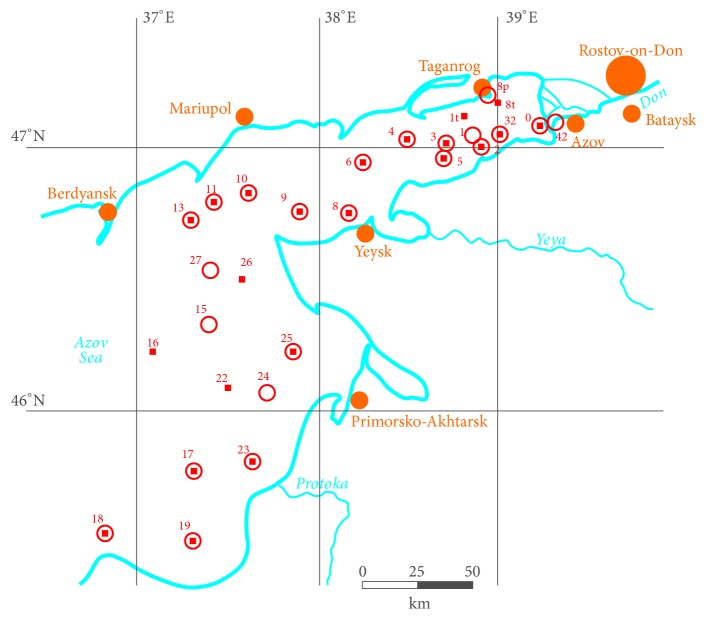
Location of sampling stations.

The present study is based on findings of field researches in the Azov Sea in 2004, 2006, and 2009 [15]. Phytoplankton and hydrochemical water sampling was undertaken with a ship bathometer from two horizons (surface and bottom layer) at monitoring stations (Figure 1). Then, the algae were precipitated on membrane filters with a pore size of 0.9 microns. Biomass was determined by the calculation method, i.e., the leveling algal cells' shape to geometric pattern and considering the specific gravity equal to unity. The total number of samples was 120. At the same time, concentrations of Mn, Co, Ni, Cr, V, Mo, Cu, and Zn were measured in the samples by the atomic absorption spectrometry method. The errors in the metal content evaluation are as follows: 0.1 *μ*g/g for Mo and Co, 3 *μ*g/g for Cu, Zn, V, and Ni, and 5 *μ*g/g for Mn.

## 3. Results

The findings from this study allow us to conclude that the intensity of the biogeochemical cycling of different metals is different. Manganese, vanadium, copper, cobalt, and molybdenum are most actively consumed (Table 1). The remaining metals are intermediate.

It is significant that a similar situation is registered in the Black Sea. According to Denisov et al., manganese (up to 1283 *μ*g / g), copper (up to 599 *μ*g/g), and vanadium (up to 80 *μ*g/g) differ by the highest concentrations in total plankton on the northeastern Black Sea shelf [16, 17]. On the northwestern shelf, despite lower metal concentrations, the leading positions are kept by copper (up to 33 *μ*g/g), manganese (average 6 *μ*g/g), and zinc (up to 72 *μ*g/g) [17].

It is determined that phytoplankton's productivity in the Taganrog Bay was 2.5 million tons, and it was 26.0 million tons of dry matter in the Azov Sea.  Table 2 presents data on the annual consumption of phytoplankton micronutrients in the Azov Sea.

To define the role of phytoplankton in the formation of the chemical constitution of bottom deposits and its participation in the geochemistry of sedimentogenesis the data given in Table 3 are used. These data show that the amount of some elements annually consumed in the Azov Sea is up to 75% of their maximum delivery together with terrigenous material. These values are the highest for vanadium and zinc, and these are the lowest for manganese and nickel. According to L.I. Tolokonnikova [18], only 17.5% of the organic matter produced by phytoplankton is precipitated on the sea bottom, and, therefore, almost the same portion of chemical elements associates with it. Of this amount, only about 1/4 remains in the bottom deposits after the mineral formation, whereas the most of the organic substances are oxidized and elements in algae go into solution enriching the entrapped water. According to the measurements, the average amount of organic substances in sediments is 5.1%; based on these data, the disposal of some chemical elements in bottom deposits is calculated.

Tikhomirov and Lukashin [19] experimentally established that elements are partially removed from water through sorption in addition to bioassimilation. As our studies has shown, biological absorption is the leading absorption process for some metals (Mn), whereas for the others metals (Co, Zn) it is sorption. Elements associated with phytoplankton after its death are included into the organometallic components' composition. These components are not completely destroyed by the decomposition of cells and, according to the findings; even a month later the most of them (60%) remain associated with dissolved and suspended organic substance [20]. There may be another approach to evaluation of role of phytoplankton in the geochemistry of sedimentogenesis. It can be the technique proposed by Strakhov [21]. Its essence is as follows. Element's concentration in biological objects is multiplied by the organic content (for phytoplankton) or CaCO_3_ (for mollusks) in sediments, and then the result is divided by the average element concentration in bottom deposits. As this can be seen from the represented data, the phytogenic share in the chemical composition's formation on the Azov Sea bottom is small (Table 4). It is most significant for vanadium (5.1) and copper (4.5)

## 4. Discussion

Different degrees of metal consumption can be an indirect indicator of the possibility of extracting some of them from suspended material through the dissolving acidic secretions of sorbed microelements' cells. [21, 22], and the other authors proposed such a way of extraction. Probably, this process takes place most intensively during periods of algal nuisance when the water mass is depleted in dissolved chemical elements. It should be also mentioned that the influence of biota on the biogeochemical cycling can not only be direct [23–27] when metals are assimilated from solutions and suspensions, but also indirect when phytoplankton growth is accompanied by hydrochemical changes in the habitat. Such a phenomenon can lead to either acceleration or to slowing of geochemical processes.

As the concentration in phytoplankton of the Azov Sea decreases, chemical elements are distributed as follows:

(1) $$\begin{array}{c} Mn>Zn>Cu>V>Cr>Ni>Co>Mo \end{array}$$

For the Black Sea, a similar sequence of microelement concentrations is established differing in the highest levels of content in plankton [17], which may serve as an evidence of the close geochemical conditions in the entire Azov-Black Sea basin:

(2) $$\begin{array}{c} Mn>Zn>Cu>Cr>V \end{array}$$

At the same time, the distribution of metals in the plankton of the Caspian Sea, which is distinguished by somewhat different conditions, is characterized by similarities with the Azov-Black Sea biogeochemical peculiarities. This may be explained by the intensity of chemical elements' consumption by algae determined by physiological functions, and the metal content indicates their importance in the metabolism of organisms. On the left side of the rows are Mn and Zn, which are chemical elements, mostly essential for photosynthesis. Vanadium also belongs to this group of elements, but its relatively insignificant extraction by phytoplankton can be explained by low concentrations of the element in water. Copper, which sustains the most important metabolic functions, occupies a central position. And close in the series are Ni, Co, and Mo, which are elements necessary for nitrogen metabolism [28].

The importance of microelements for various physiological and biochemical processes in living organisms has been noted by many authors. The works of [29, 30] are devoted to studying the factors of metals' accumulation in plankton organisms in the Azov Sea. These authors found significant correlation (the correlation coefficients range within 0.50–0.78) between the suspended and dissolved forms of copper, zinc, manganese, and nickel in the Azov Sea and alkaline phosphatase activity and sestone esterases, which are key enzymes for phosphorus and carbon turnover and cell metabolism rates. It is specific that during the period of the noted studies [29, 30] the concentration of copper in water in all samples exceeded the threshold limit value; for zinc, manganese, and nickel, these values were 83%, 8%, and 21%, respectively, and, apparently, these concentrations were not limited to these species of living organisms of the Azov Sea ecosystem.

At the same time, the metabolic activity of hydrolytic extracellular enzymes of plankton communities is significantly affected by water pollution with mercury. Thus, the analysis of the relationship between the content of mercury in water and the activity of nonspecific esterase and alkaline phosphatase of plankton performed earlier by Fedorov et al. [31] in the Don's delta showed the toxic effects of this metal, as evidenced by negative correlation coefficients.

The study by Rozhanskaya [32] allowed establishing the relationship between the content of elements in phytoplankton, the hydrochemical regime of the reservoir, and the taxonomic composition of algae. This can explain the selective extraction of certain chemical elements by various microphytes from seawater and suspension.  Table 5 shows the metal-depleted pure culture* of Coscinodiscus jonesianus* and blue-green algae. A mixed complex of diatoms consisting mainly of* Thalassionema nitzschioides* and* Microcystis aeruginosa* is also enriched in microelements.

The elements in the “river-sea” transitional areas are assimilated most intensively. The activity of the relevant process is facilitated by the not only arrival of dissolved forms with river runoff, but also the high productivity [33, 34] and the taxonomic (at the level of species) composition of phytoplankton having significant ability to consume. As a result, in the Taganrog Bay, of which capacity is only ~8% from the capacity of the Azov Sea, almost the same number of bioelements is involved in the biogeochemical cycling as in the rest of the sea. A similar situation is observed in the Caspian Sea, within which the most intensive assimilation is usual in its northern part influenced by the discharge of the Volga, Ural, and Terek rivers, as well as in some near river mouths of the Black Sea. Such a situation develops, for instance, in the region of the Inguri River mouth [17]. Thus, the participation of different types of algae in the biogeochemical cycling of elements would not be the same. All this determines the inconstancy of the chemical composition of phytoplankton, and the recorded seasonal and annual fluctuations are naturally associated with the changing habitat and the constant change of phytoplankton assemblages.

The results show the highest concentrations of Mn, Ni in 2009, for which a high amount of water discharge of the Don River and intake of great masses of biogenic components and microelements were typical. Mn and Ni are elements necessary for functioning of living organisms. The amounts of their delivery to the Azov Sea (Taganrog Bay) together with the river discharge in the content of live and dead phytoplankton are about two times lower than in the ion form. Evidently, the influence of the anthropogenic factor in 2009 (for instance, discharge of coal shaft waters or metal processing near the sea basin) was stronger than in the previous year of observation. The accumulation of metals in algae was facilitated by water conversion in the Taganrog Bay by extensive development of the brackish-water assemblage, as well as by intensive process of sulfate reduction, which led to the active migration of metals in the sediment-water system [34–37]. Low content of elements in phytoplankton in 2004 were caused by extremely unfavorable habitat conditions, i.e., the high degree of salinity, the low freshwater supply, and the soft concentration of nutrients in the water layer.

Strakhov [21] showed that the size of reservoirs acts as an important geochemical factor. Morphometric indexes affect the course and the intensity of biochemical processes. This is proven by the examples of the Azov and Caspian seas, which differ from one another by of area and depth. It is enough to note that, despite the proximity of the biochemical structure and the similarity of the species spectrum [38], the intensity of consumption per volume unit of the Caspian phytoplankton is lower than in the case of the Azov Sea (Table 2). This seems to be natural if to consider that photosynthesis in the Azov Sea embraces the entire water layer, and it embraces the only upper part of the water layer in the Caspian Sea. In addition, water bodies differ by different degrees of river discharge influence on biogeochemical processes. For example, the Azov Sea ecosystem, for which the average long-term natural river discharge was just over 12% of the reservoir volume, is characterized by extraordinary productivity of all components, especially of phytoplankton. In the Caspian Sea, the continental runoff is lower 0.3%, and it affects mainly its northern part. Another inference is as follows: together with increase in water bodies' size, the areas of river discharge influence on biogeochemical processes are reduced and the significance of the transition areas of the “river-sea” system in the biological cycling of elements decreases. This is also confirmed by the data obtained earlier for the Black Sea. It was estimated by Denisov and Chernousov that the amount of heavy metals annually associated with the Black Sea's total plankton (its production is 170 million tons, which is in several times higher than in the Azov Sea) is 3 thousand tons for vanadium, 5.6 thousand tons for chromium, 22.1 thousand tons for copper, 101.4 thousand tons for manganese, and 41.6 thousand tons for zinc [17]. It is significantly lower than in the Azov Sea (Table 2), and it will be even lower if to separate the proportion of metal content in zooplankton.

According to our data, a high turnover ratio is typical for some trace elements. So, it reaches 9-10 in vanadium, 2-3 in manganese, and 0.1-0.5 in copper, nickel, molybdenum, and cobalt. Consequently, the Azov Sea is characterized by both high rates of biogeochemical cycling and significant consumption of the majority of trace elements.

## 5. Conclusions

1. Thanks to its ability to develop quickly in large quantities, phytoplankton plays active role in the biogeochemical cycling of elements.
2. The annual consumption of chemical elements by algae is inferior to supplying them with terrigenous material. However, under the influence of biotic and abiotic factors, the most of the organic matter produced by algae and elements connected with these are destroyed and dissolved directly within the water column reentering the biogeochemical cycling.
3. A few metals reach sea bottom with the rest of planktonic algae, but even there, as a result of organic-matter degradation, elements are released and transferred to entrapped water. The lowered maintenance of minerals in bottom deposits in relation to the weighed substance is interpreted.
4. In the matrix of bottom deposits, a small part of the amount extracted by phytoplankton remains, and this may lead researchers to the wrong conclusion about the secondary role of microalgae in chemical processes in sediments.

## Figures and Tables

**Table 1:** Interannual changes in average trace elements content in total phytoplankton of the Azov Sea, *µ*g/g of dry weight.

Elements	2004	2006	2009
Mn	52.0	250.0	280.0
Ni	17.0	40.0	45.0
Co	5.0	4.0	-
Cr	42.0	67.0	56.0
V	30.0	103.0	30.0
Mo	1.0	1.3	1.0
Cu	85.0	93.0	48.0
Zn	95.0	130.0	110.0

**Table 2:** The annual consumption of phytoplankton micronutrients in the Azov Sea.

Elements	Taganrog Bay			Open sea			Entire sea		
	t	mg/Nm^2^	mg/Nm^3^	t	mg/Nm^2^	mg/Nm^3^	t	mg/Nm^2^	mg/Nm^3^
Mn	565.0	106.6	22.4	3257.0	86.2	10.0	3822.0	89.2	10.8
Ni	71.3	13.5	2.8	279.7	7.4	0.9	351.0	8.3	1.1
Co	10.5	2.0	0.4	20.8	0.6	0.1	31.3	0.8	0.1
Cr	132.5	25.0	5.3	280.8	7.4	0.9	413.3	10.0	1.2
V	175.0	33.0	6.9	1255.0	22.8	2.6	1430.0	24.3	2.9
Mo	205	0.5	0.1	5.2	0.2	0.1	7.7	0.3	0.1
Cu	222.5	42.0	8.8	462.8	12.2	1.4	485.3	16.6	2.0
Zn	505.1	95.4	20.0	2614.9	69.1	8.9	3120.0	73.0	9.8

**Table 3:** Consumption of microelements by phytoplankton and their deposition with remnants of planktonic algae in the bottom deposits of the Azov Sea, t.

Elements	Delivery to the sea with terrigenous material	Consumption by phytoplankton	Precipitation with the rest of algae	Transition of algae rest during their mineralization into silt solutions	Deposition on bottom
Mn	16265,0	3822,0	668,8	473,9	194,9
V	1951,8	1430,0	250,2	177,3	72,9
Zn	4879,5	3120,0	546,0	386,9	159,1
Ni	2277,1	351,0	122,8	87,0	35,8

**Table 4:** Trace minerals in components of bottom deposits of the Azov Sea, %%.

Elements	Average content in bottom deposits, %	Content ratio		
		organic substance (mainly phytoplankton)	mollusc shell	terrigenous and authigenic components
Cr	0.007	0.4	0.2	99.4
Ni	0.006	2.6	0.9	96.5
V	0.01	5.1	0.2	94.7
Mn	0.025	3.1	17.6	79.3
Cu	0.001	4.5	21.9	73.6

**Table 5:** Distribution of microelements in various types of phytoplankton of the Azov Sea, *µ*g/g of dry weight (after [32]).

Chemical elements	Mn	Cu	Zn
*Coscinodiscus jonesianus* (almost pure culture)	40.0	16.0	310.0
Various diatoms (mainly *Coscinodiscus*-40-90%, *Thalassionema nitzschioides-*10-50%)	200.0	62.0	1000.0
Blue-green algae (mainly *Microcystis aeruginosa*-95%)	31.0	28.0	630.0

## Data Availability

The data used to support the findings of this study are available from the corresponding author upon request.
